# Integration of metabolomics and transcriptomics indicates changes in MRSA exposed to terpinen-4-ol

**DOI:** 10.1186/s12866-021-02348-2

**Published:** 2021-11-04

**Authors:** Feng Cheng, Yanan Mo, Keyuan Chen, Xiaofei Shang, Zhen Yang, Baocheng Hao, Ruofeng Shang, Jianping Liang, Yu Liu

**Affiliations:** Key Laboratory of New Animal Drug Project, Gansu Province, Key Laboratory of Veterinary Pharmaceutical Development, Ministry of Agriculture and Rural Affairs, Lanzhou Institute of Husbandry and Pharmaceutical Sciences of Chinese Academy of Agriculture Sciences, 730050 Lanzhou, People’s Republic of China

**Keywords:** Terpinen-4-ol, MRSA, Biofilm, Metabolomics, Transcriptomics

## Abstract

**Background:**

This study investigated the effects of terpinen-4-ol on *methicillin-resistant Staphylococcus aureus* (MRSA) and its biofilm, and the possible mechanisms governing this effect.

**Results:**

We observed that terpinen-4-ol has good antibacterial activity and inhibits the formation of MRSA biofilm. The MIC and MBC values for terpinen-4-ol against *S. aureus* were 0.08% ~ 0.32%. And terpinen-4-ol at 0.32% could kill all bacteria and clear all biofilms. Untargeted metabolomic and transcriptomic analyses showed that terpinen-4-ol strongly inhibited DNA and RNA biosynthesis in MRSA at 2 h after treatment by affecting genes and metabolites related to purine and pyrimidine metabolic pathways. Some differential genes which play important roles in DNA synthesis and the production of eDNA from biofilm exposed to terpinen-4-ol was also significantly decreased compared with that of the control.

**Conclusions:**

Terpinen-4-ol has good antibacterial activity and significantly inhibits the formation of MRSA biofilm by inhibiting purine and pyrimidine metabolism.

## Background

In recent years, essential oils have become widely used in the biomedical, cosmetics and food industries and other fields due to their broad antibacterial activity and few side effects [1]. Numerous essential oils can not only effectively inhibit the growth of microorganisms but also clear microbial biofilms; such as TTO [2], litsea cubeba oil [3], oregano oil [4] and clove oil [5]. TTO is a colorless to light yellow liquid extracted from the fresh branches and leaves of *Melaleuca alterniflora* in Australia by steam distillation, with the smell of camphor and cooling sensation of menthol [6]. TTO is known for its antiseptic, antimicrobial, and anti-inflammatory properties [7].

Terpinen-4-ol was considered to be the main active ingredient of TTO [6], and ISO 4730-2017 stipulates that the content of terpinen-4-ol should not be less than 30% [8]. But there were few studies about the strong antibiofilm activity [9] of terpinen-4-ol, and its mechanism was not yet fully understood.

*Staphylococcus aureus* (*S. aureus*) is one of the earliest described pathogens in humans and one of the most common causes of infection [10]. Due to the overuse of antibiotics in treating human and animal diseases, drug resistance in *S. aureus* has become increasingly serious and has led to the MRSA pandemic [11]. MRSA causes many diseases and conditions, such as nosocomial infections, pneumonia, sepsis, and skin infections. Studies have shown that the formation of MRSA biofilm is a main factor contributing to the increased drug resistance and toxicity of this pathogen [12]. Biofilms are communities of microorganisms formed by many bacteria that allows them to adhere to a surface, and it can serve as a barrier for bacteria against host immune cells [13]. The bacteria in biofilm are reportedly approximately 1000 times more resistant to antibacterial agents than are planktonic bacteria [14]. Currently reported methods for the treatment of biofilm of S.aureaus include quorum-sensing modulators, Bioactive Molecules Interfering with Staphylococcal Biofilms and Bacteriophage-Based Antibiofilm Strategies [15]. Antibiotics, AMP and plant extracts are commonly used in the treatment of biofilm infection. The anti-MRSA biofilm activity of terpinene-4-ol was studied in this paper [16] .

## Results and discussion

### Antibacterial activity

**Fig. 1:**
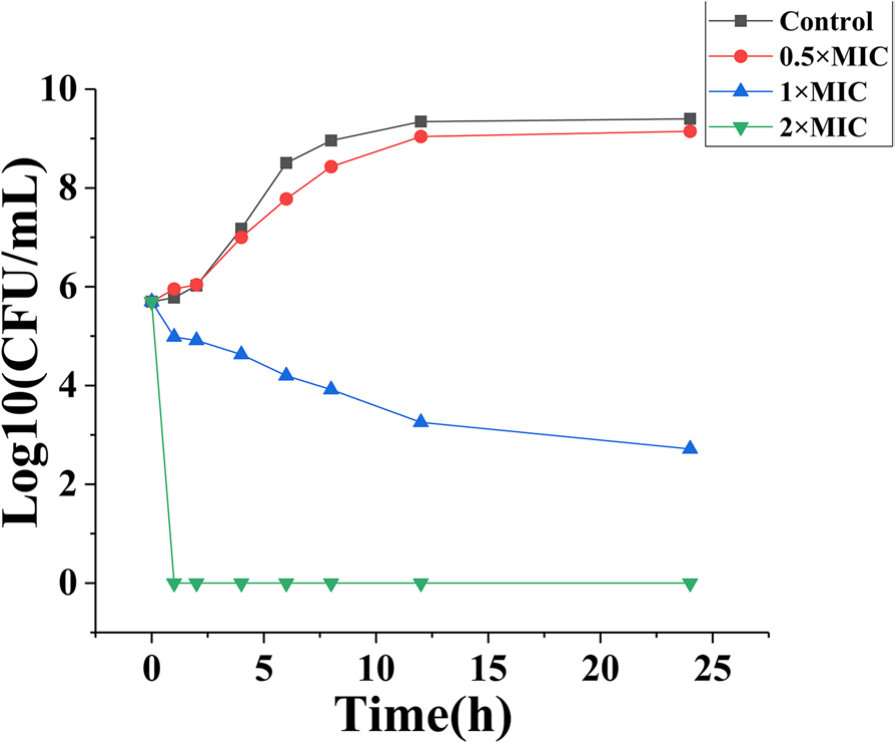
Time-killing curves of terpinen-4-ol against MRSA

TTO is a potential anti-bacterial [7, 17] and anti-fungal product [18, 19], but there were few studies about the anti-MRSA activity of terpinen-4-ol, a main ingredient of TTO. Our research focused on the antibacterial activity of terpinen-4-ol against MRSA. The MIC and MBC values of terpinen-4-ol against MRSA were 0.16 and 0.32%(v/v) while it against other *S. aureus* were 0.08% ~ 0.32% (Table 2), which is similar to Loughlin’s result (0.25%) [20]. In Table 1, we could see the MBC value was two times the MIC value, which leads us to know that terpinen-4-ol exhibited bactericidal properties against MRSA and other strains in this study [21]. In addition, a time-kill curve of MRSA exposed to terpinen-4-ol was generated by performing plate counts (Fig. 1). We found that 0.32% terpinen-4-ol killed all the bacteria within 2 h and that terpinen-4-ol at 0.16% or 0.08% could obviously inhibit the growth of MRSA, which also showed terpinen-4-ol exhibited bactericidal properties against MRSA.

**Table 1:** Antimicrobial activities of terpinen-4ol against 13 *S. aureus* strains

Bacteria	MIC(v/v)	MBC(v/v)
ATCC 43300	0.16%	0.32%
S-1	0.08%	0.16%
S-2	0.08%	0.16%
S-3	0.16%	0.32%
S-4	0.08%	0.16%
S-5	0.32%	0.32%
S-6	0.08%	0.16%
S-7	0.16%	0.16%
S-8	0.08%	0.16%
S-9	0.16%	0.16%
S-10	0.32%	0.32%
S-11	0.16%	0.32%
S-12	0.16%	0.32%

### Antibiofilm activity

**Fig. 2:**
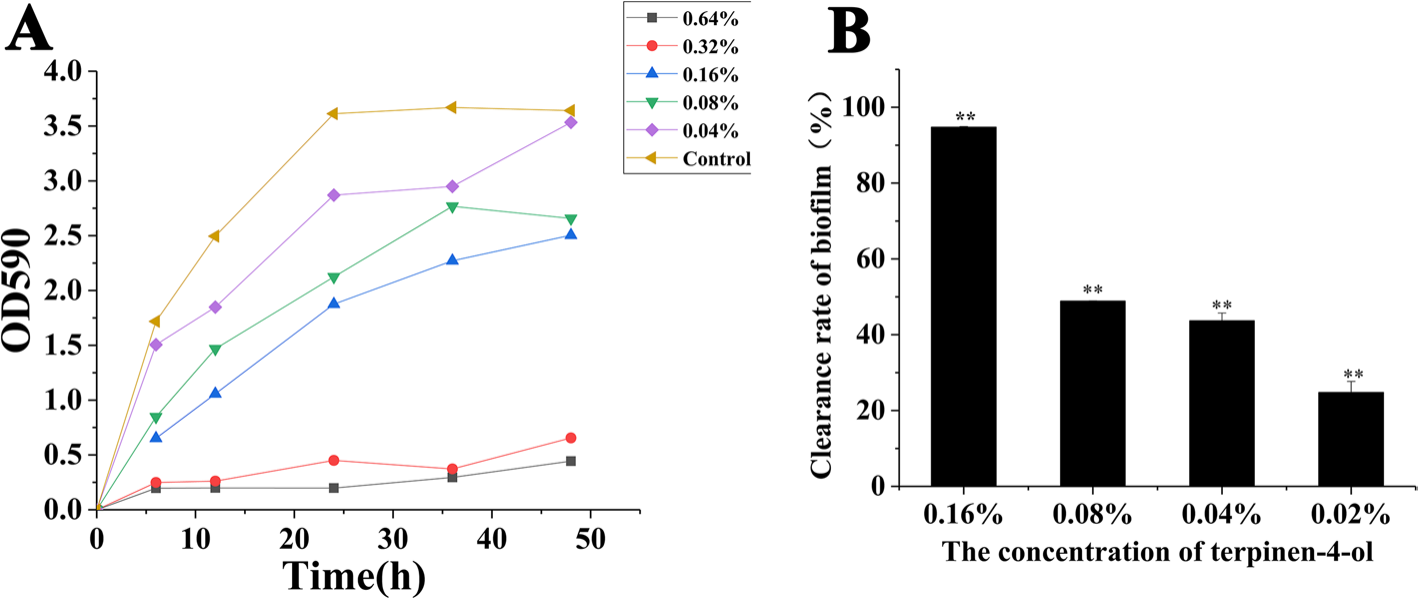
Effect of terpinen-4-ol on MRSA biofilms, **A** Effect of terpinen-4-ol on the formation of MRSA biofilms, the ordinate represents the relative content of MRSA biofilms. **B** Effect of terpinen-4-ol at various concentrations on the mature biofilm of MRSA, the ordinate represents the removal rate of terpinen-4-ol on MRSA biofilms. **: Compare with blank control group, *P* < 0.01, t test

The antibiofilm activity of terpinen-4-ol was also assessed by the crystal violet staining. As shown in Fig. 2A., there was almost no MRSA biofilm formation under treatment with terpinene-4-ol at concentrations of 0.64 and 0.32%. Terpinene-4-ol at 0.16% also had a significant effect on the formation of MRSA biofilm, and the inhibition rates at 24 h and 48 h were 48.09% ± 0.97% (*P* < 0.01) and 31.20% ± 2.13% (*P* < 0.01). It can be seen that higher concentrations of terpinen-4-ol effectively inhibited the formation of biofilm.

In this study, terpinen-4-ol behaved its inhibition in MRSA biofilm formation. It also confirmed other authors’ results that terpinen-4-ol could destroy the biofilm of many oral pathogens such as, *Streptococcus mutans*, and *Lactobacillus acidophilus* [22] *Porphyromonas gingivalis*, *Fusobacterium nucleatum* [23]. We could see that terpinen-4-ol could inhibit biofilm formation of MRSA in a concentration-dependent manner. With the decrease of concentration of terpinen-4-ol, the inhibition rates gradually down.

**Fig. 3:**
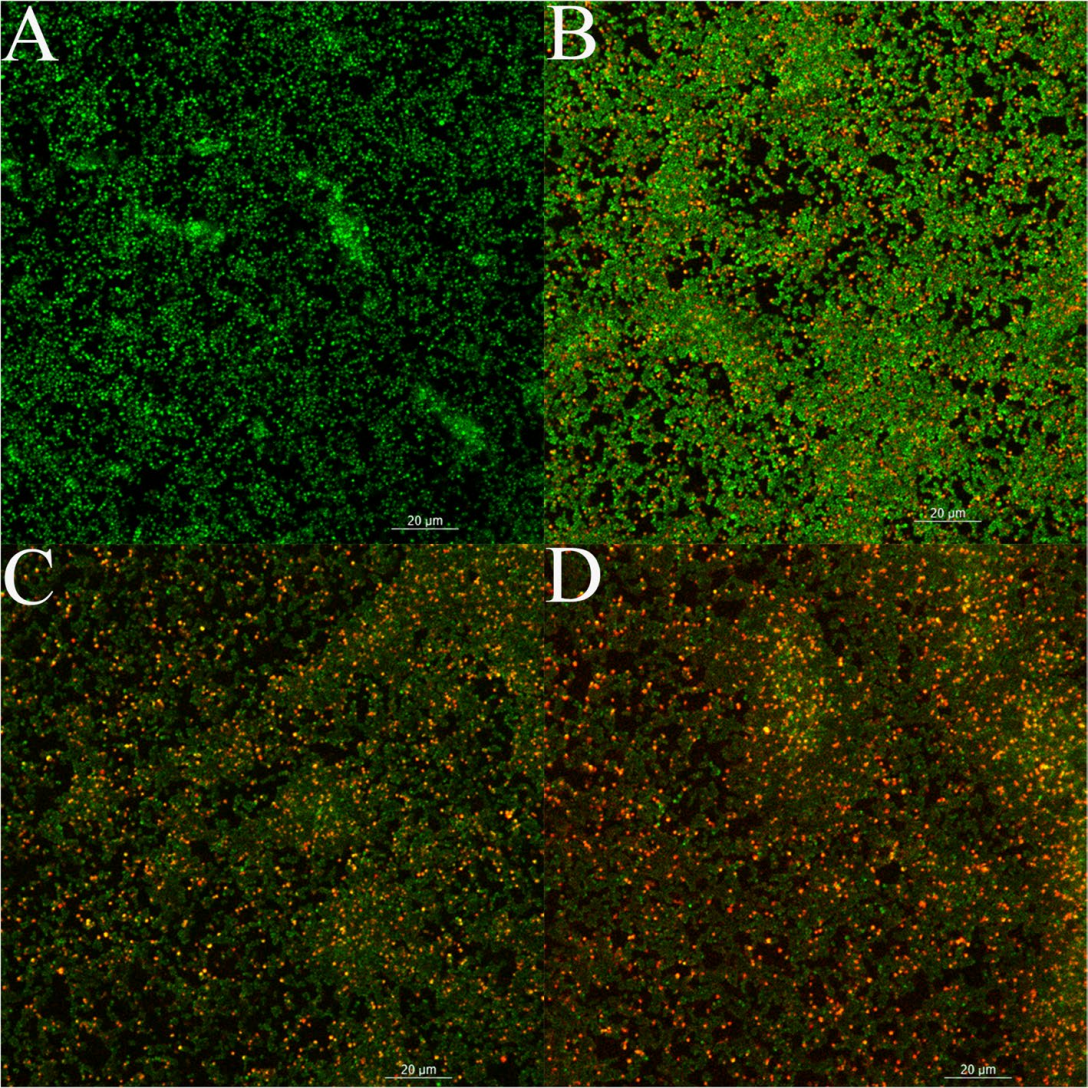
CLSM image of LIVE/DEAD stained MRSA biofilm grown on cell slide. Green (viable cells) and red (dead cells). **A** Control cells (no treated). **B** Cells treated with terpinen-4-ol (0.04%). **C** Cells treated with terpinen-4-ol (0.08%). **D** Cells treated with terpinen-4-ol (0.16%)

The destruction of biofilms by terpinen-4-ol was also evaluated. As shown in Fig. 2B, 0.16% terpinene-4-ol achieved a clearance rate of 93.90% ± 3.33% (*P* < 0.01) for MRSA mature biofilm. As the concentration of terpinen-4-ol decreased, the clearance rate gradually decreased. The viability of the preformed MRSA biofilm exposed to terpinen-4-ol was also verified by CLSM analysis. As shown in Fig. 3, as the concentration of terpinen-4-ol increased, the amount of red fluorescence (indicating dead bacteria) increased while that of green fluorescence (indicating live bacteria) decreased.

Terpinen-4-ol showed its activity in clearing MRSA biofilm. Generally, it is more difficult to remove mature biofilm than to inhibit its formation. Thus, terpinen-4-ol could consider to be a potential antibiofilm agent, for it can not only inhibit the formation of biofilms, but also destroy mature biofilm. Therefore, it is necessary to further study the anti-biofilm activity of terpinene-4-ol and explain its mechanism of action.

### Antibiofilm mechanism

#### Transcriptomics

**Fig. 4:**
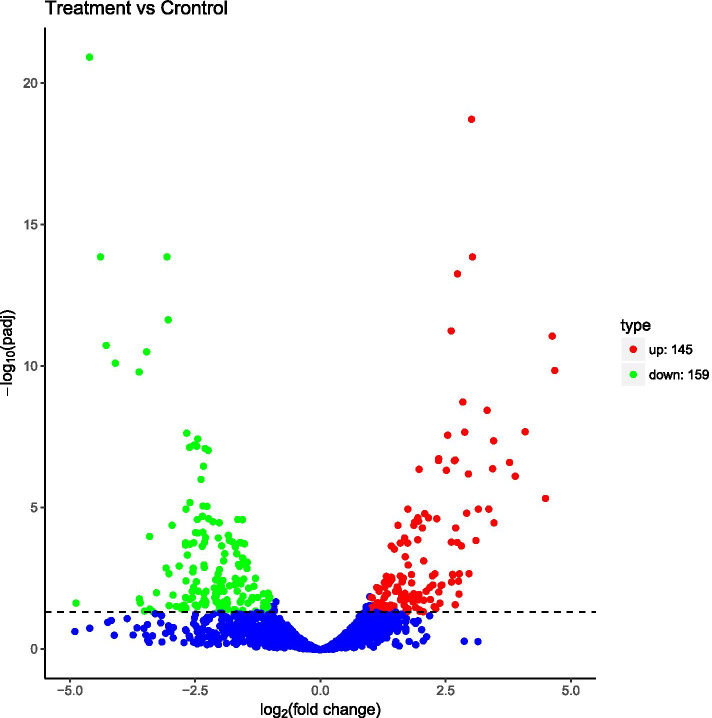
Volcano plot of DEGs in MRSA treated with terpinen-4-ol

To understand the changes in MRSA due to treatment with terpinen-4-ol, we measured the transcriptome of MRSA exposed to terpinen-4-ol and screened 304 DEGs as defined by FC > 2 and Q < 0.05. Among these DEGs, 159 genes were downregulated and 145 genes were upregulated in terpinen-4-ol-treated relative to control (Fig. 4). The RNA-seq data were submitted to Gene Expression Omnibus (GEO) under accession number GSE157638.

**Fig. 5:**
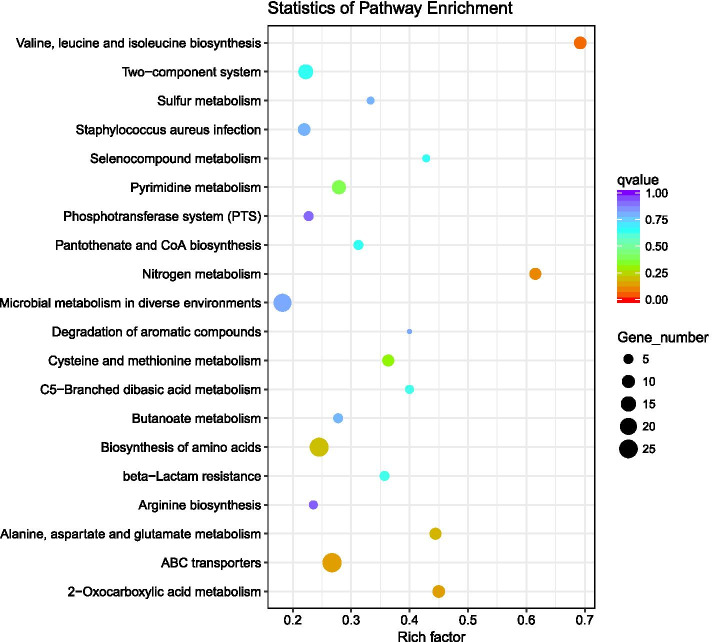
Bubble chart of KEGG enrichment results of transcriptome

KEGG pathway analysis was used to obtain insights into the biological functions of the DEGs (Fig. 5). Compared with the control group, there were some pathways different, including valine, leucine, and isoleucine (branched chain amino acids, BCAAs) biosynthesis. Nitrogen metabolism, ABC transporters, 2-oxocarboxylic acid metabolism and etc., were significantly enriched (*P* < 0.05) in terpinen-4-ol-treated group. Some of those pathways related to the formation of MRSA biofilm. For example, BCAAs, synthesized by 2-oxocarboxylic acid, regulate the biosynthesis of bacterial amino acids [24] and nucleotide metabolism [25] by combining with CodY, and they can also regulate the biofilm of S.aureus [26–28]. There were 9 genes related to 2-oxocarboxylic acid and BCAAs have been down in MRSA treated with terpinen-4-ol, which showed us the BCAA metabolic pathway of MRSA may be affected by terpinen-4-ol. Fig. 4.

Furthermore, there were other pathways that warrant our attention, such as purine metabolism, quorum sensing (QS), and β-Lactam resistance. Most of these pathways are related to the synthesis of nucleic acids or ATP, which are important to biofilm formation of MRSA. We then used metabolomics to verify the results of the transcriptomics analysis.

#### Metabolomics

**Fig. 6:**
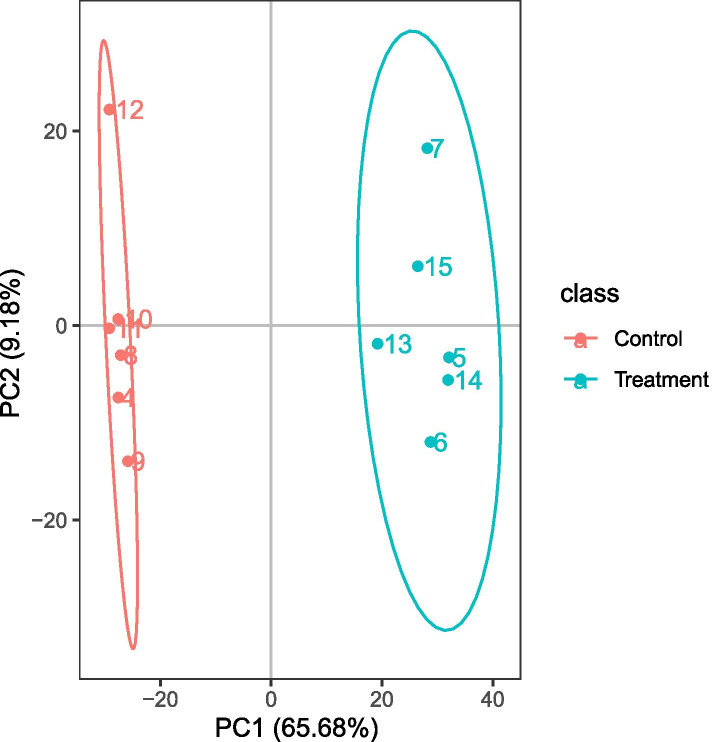
PCA plot

**Fig. 7:**
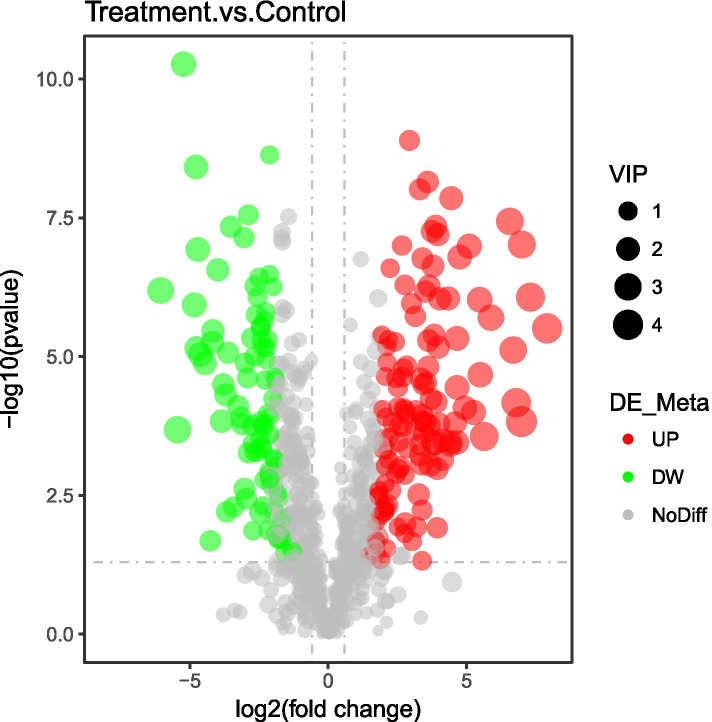
Volcano plot of different metabolites in MRSA treated with terpinen-4-ol

**Fig. 8:**
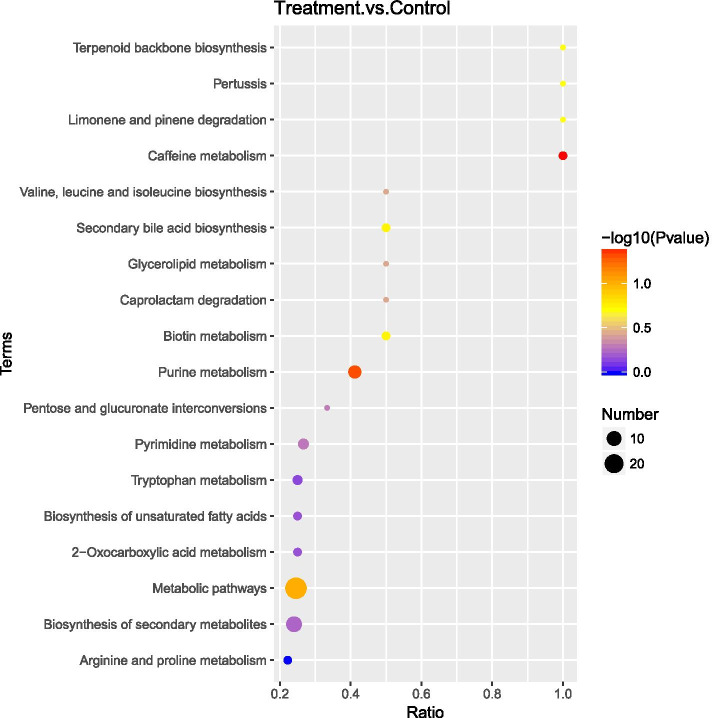
Bubble chart of KEGG enrichment results

Twelve sets of data from the six biological replicates were analyzed to determine the changes in metabolites that occur in MRSA following treatment with terpinen-4-ol. PCA analysis (Fig. 6) showed that the quality of data was high. Two hundred fifteen differential metabolites were screened based on FC > 1.5 or FC < 0.67, *P* < 0.05, and VIP value > 1.0. Among these differential metabolites, 125 were upregulated and 90 were downregulated in terpinen-4-ol-treated MRSA relative to control MRSA (Fig. 7). Eighteenenriched pathways (Fig. 8) were identified by KEGG enrichment analysis, including caffeine metabolism (*P* < 0.05). Caffeine can be converted to purine, and we found that many intermediate products of purine metabolism and the contents of its constituents have decreased, including xanthine and xanthosin, which are intermediate products of purine metabolism, guanosine, and adenosine, which are components of purine metabolism, 2′-deoxyadenosine, and deoxyadenosine, which are components of DNA.

In addition, there were also some metabolites related to pyrimidine metabolism have decreased. The content of these substances is closely related to DNA synthesis, and the reduction of their content may lead to a reduction in the amount of bacterial DNA. Thus, we conjectured that terpinen-4-ol achieves its anti-MRSA activity by inhibiting the synthesis of DNA.

#### eDNA quantification

**Fig. 9:**
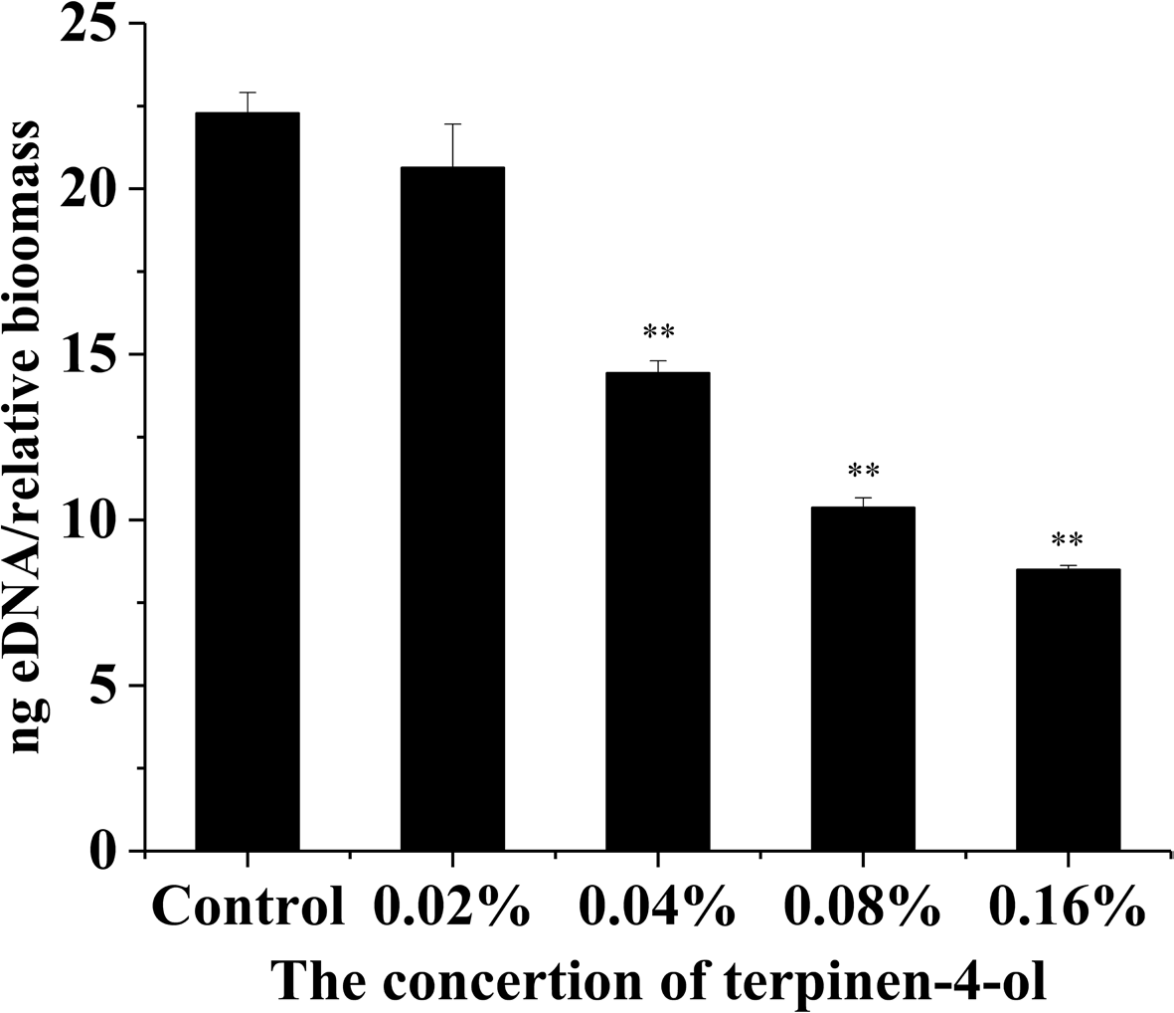
Effect of terpinen-4-ol at various concentrations on eDNA of MRSA biofilms. **: Compare with blank control group, *P* < 0.01, t test

eDNA an important component of the EPS released by the lysis of some bacteria during the formation of the biofilm [29], and it plays a vital role in all stages of biofilm formation, including initial bacterial adhesion, aggregation, microcolony formation, and determining the structure of biofilm [30]. The amount of eDNA in the medium of the biofilm was measured by spectrophotometer and reported as the eDNA per relative biomass to account for the number of bacteria in the biofilm. The production of eDNA from biofilm exposed to terpinen-4-ol was significantly decreased compared with that of the control (Fig. 9). In particular, the eDNA content of the biofilm treated with 0.16% terpinen-4-ol was decreased relative to the control content by 61.88% ± 0.58%. The decrease in the content of eDNA reduces the adhesion ability between bacteria, leading to the destruction of the integrity of its biofilm. Thus, we inferred terpinen-4-ol inhibited biofilm formation of MRSA by inhibiting its release of eDNA.

#### Q-RT-PCR

To verify our results, we also performed qRT-PCR verification of some of the differential genes, which play important roles in DNA synthesis (Table 2). In terpinen-4-ol-treated MRSA relative to control MRSA, the expression of *deoD* was upregulated by 12.378 ± 0.541 times, and the expression of *pyrB* was downregulated by 3.049 ± 0.147 times. Those results were largely consistent with the transcriptome results that terpinen-4-ol inhibit purine and pyrimidine metabolism.

**Table 2:** Selected MRSA genes that displayed altered expression after terpinene-4-ol treatment of biofilm as determined by RNA-seq and real-time RT–PCR

Gene	Description	Fold Change ± SD	
		qRT-PCR	RNA-seq
*carB*	carbamoyl phosphate synthase large subunit	−2.054 ± 0.304	−6.36
*arcC*	carbamate kinase	+ 2.371 ± 0.051	3.38
*deoD*	purine nucleoside phosphorylase	+ 12.378 ± 0.541	24.723
*pyrF*	orotidine 5′-phosphate decarboxylase	−1.297 ± 0.697	−5.07
*pyrB*	aspartate carbamoyltransferase catalytic subunit	−3.049 ± 0.147	−5.45

#### Conjoint analysis

**Fig. 10:**
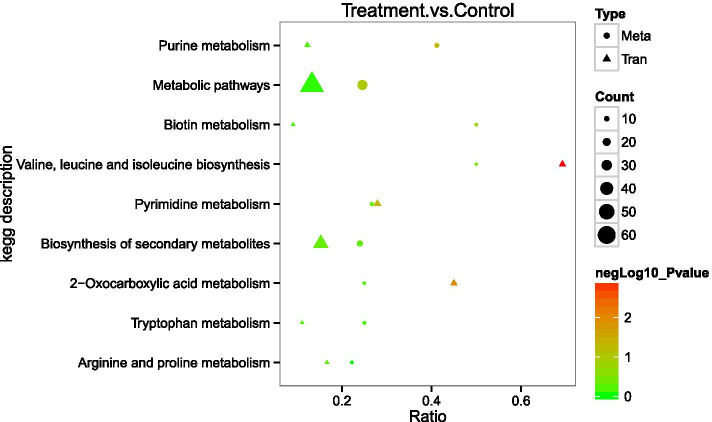
KEGG enrichment of conjoint analysis

The differential genes identified from the transcriptomics analysis and the differential metabolites identified from metabolomics analysis were represented in the KEGG pathway database (Fig. 10). After the enriched pathways were identified for the differential genes and metabolites separately, it was found that the differential genes and metabolites were mainly involved in the synthesis of MRSA nucleic acid. In the transcriptome results, 16 genes related to nucleic acid synthesis were altered, including *nrdF*, *arcC*, *deoD*, *pyrF*, *pyrB* and *carB*. In the metabolome results, the contents of 11 metabolites related to nucleic acid synthesis, including xanthine, 2′-deoxyadenosine, inosine, cytosine, thymidine, and deoxyadenosine, were significantly reduced in terpinen-4-ol-treated MRSA relative to control MRSA.

ATP is composed of adenine, ribose and 3 phosphate molecules, and is the most direct source of energy in living organisms [31]. The reduction of purine and pyrimidine synthesis materials in terpinen-4-ol-treated MRSA affects its ATP synthesis, resulting in different degrees of inhibition of energy metabolism and membrane transport systems. In transcriptome and metabolome analysis, we found that many ATP-related genes and small molecule metabolites were affected by terpinen-4-ol treatment to varying degrees, especially ABC transporters and energy metabolism related genes. ABC transporter is the main transporter of MRSA efflux pump, which is closely related to the formation of MRSA biofilm and the quorum sensing system [32, 33]. The decrease of efflux pump activity can inhibit the formation of bacterial biofilm [34]. The ABC transporter is also involved in one of the main mechanisms of MRSA drug resistance. A study has shown that the combination of terpene-4-ol and β-lactam antibiotics has a significant synergistic inhibition effect on MRSA. In addition, TTO can inhibit the growth of Botrytis cinerea by inhibiting its energy metabolism [35]. Therefore, we speculate that terpinen-4-ol can inhibit the growth of PMRSA by inhibiting the synthesis of purines and pyrimidines.

The biosynthesis of pyrimidine nucleotides can affect biofilm by influencing the expression of the genes csgDEFG [36]. The interplay between the rescue synthesis pathway of nucleotides and Curli amyloid fibers’ generation may be closely related to cellulose and other EPS responses to environmental pressure [37–39]. *pyrB* and *carB* are the key enzymes of the nucleotide rescue pathway, being very important for the rescue synthesis of nucleotides. In our research, the expression of these two genes was inhibited by terpinen-4-ol treatment. Furthermore, the levels of four nucleotides, the raw material for UMP synthesis, declined significantly after terpinen-4-ol treatment, indicating that the salvage synthesis of pyrimidine nucleotides was inhibited by terpinen-4-ol. The inhibition of pyrimidine nucleotides might block the production of modified nucleotides acting as signal molecules for biofilm formation, such as c-di-GMP [40], which is an important signal molecule in biofilm regulation. In addition, the inhibition of pyrimidine nucleotides biosynthesis might lead to the inhibition of eDNA synthesis, which could lead to the destruction of biofilm. The observed decrease in eDNA production in terpinene-4-ol-treated MRSA relative to control MRSA supports this possibility. Therefore, we hypothesized that terpinene-4-ol may inhibit the formation of MRSA biofilm by inhibiting the pyrimidine metabolism of MRSA, but this needs to be further verified.

## Conclusion

Terpinen-4-ol has good antibacterial activity and significantly inhibits the formation of MRSA biofilm. It demonstrated the excellent potential in anti-biofilm drug. The results of the transcriptomics and metabolomics analyses suggest that terpinen-4-ol inhibits the growth of MRSA by inhibiting purine and pyrimidine metabolism. According to the decrease of eDNA in MRSA biofilm, it is further speculated that the mechanism of terpinen-4-ol inhibiting the formation of MRSA biofilm may also inhibit the synthesis of purines and pyrimidines. These findings provide new insights into the antibacterial mechanisms of terpinen-4-ol and may promote the study of the pharmacological activity of terpinen-4-ol as a natural antibacterial product.

## Materials and methods

### Materials

MRSA (ATCC 43300) was obtained from Beina Biotechnology (Beijing, China). Twelve isolates of *S. aureus* were obtained from the manure of the dairy farm in Gansu China, and were labeled S-1 through S-12. MHA and MHB were obtained from Aobox Biotechnology (Beijing, China). TSB and BHI were obtained from Oxoid (Fisher, UK), and terpinen-4-ol was obtained from Sigma Aldrich (Shanghai, China).

### Testing the susceptibility of planktonic Bacteria

The MIC of terpinen-4-ol was determined according to the Clinical and Laboratory Standard Institute (CLSI) guidelines. One standard strain and 12 clinical strains of *S. aureus* were tested in this study.

### Time-kill curve

Time-kill curve of terpinen-4-ol against MRSA strain ATCC 43300 was generated. MRSA was cultivated in MHB with terpinen-4-ol and incubated at 37 °C. The number of MRSA was measured by the plate counting method at regular time intervals.

### Assays the effect on biofilm

The adhesion of biofilm was measured by crystal violet staining and CLSM. The crystal violet staining method was performed according to a previous report and used to determine the inhibition and clearance of MRSA biofilm by terpinen-4-ol at different concentrations. For the effect of terpinen-4-ol on the removal of biofilms, a mature biofilm is first cultivated, and then different concentrations of terpinen-4-ol was added. After a certain period of time, the amount of biofilm is determined by crystal violet. The biofilm was cultured and measured following Srdjan Stepanović’s report [41].

CLSM was performed by staining a pre-established biofilm with the LIVE/DEAD BacLight Bacterial Viability kit (Invitrogen Molecular Probes, Carlsbad, USA) according to the manufacturer’s instructions. CLSM images were captured using an LSM 800 (ZEISS, LSM 800) with a 40× objective lens. ZEN 2.3 was used to analysis and exporting images of CLSM.

### RNA-seq and enrichment analysis

MRSA was exposed to terpinen-4-ol at a concentration of 1/2 × MIC (0.08%, v/v) for 2 h, and 3 treatment samples and 3 control samples were analyzed. Total RNA was extracted from MRSA using an RNAprep Pure Bacteria Kit (Tiangen, Beijing, China). The transcriptome sequencing and analysis were conducted by Novogene (Tianjin, China) using the Illumina sequencing platform (Illumina NovaSeq). DEGs screening and KEGG enrichment analysis respectively used Bowtie v2.2.3 and KOBAS.

### Metabolite extraction and analysis

MRSA was exposed to terpinen-4-ol at a concentration of 1/2 × MIC (0.08%, v/v) for 2 h, and 6 treatment samples and 6 control samples were analyzed. MRSA (100 mg) were individually grounded with liquid nitrogen and the homogenate was resuspended with prechilled 80% methanol and 0.1% formic acid by well vortexing. Take an equal volume sample from each experimental sample and mix it as a quality control sample for quality control to ensure the quality of the final collected data. And the metabolites analysis was carried out to analyze using a Vanquish UHPLC system coupled with an Orbitrap Q Exactive series mass spectrometer (Thermo Fisher, USA). The raw data files generated by UHPLC-MS/MS were processed using the Compound Discoverer 3.1 (CD3.1, Thermo Fisher) to perform peak alignment, peak picking, and uantitation for each metabolite. MetaX software [42] was used for logarithmic conversion and centralized formatting of the data, and then PCA analysis is carried out. The Variable Importance in the Projection (VIP) value of the first principal component of the PLS-DA model is used. The VI*P* value represents the contribution rate of the difference in metabolites in different groups; the Fold Change (FC) is for each metabolism The ratio of the mean value of the repeated quantitative values of all organisms in the comparison group; combined with the P value of T-test to find the differentially expressed metabolites, set the threshold value to VIP > 1.0, and the multiple of difference FC > 1.5 or FC < 0.667 and *P* value < 0.05, screen out the different metabolites.

### eDNA quantification

The extraction of eDNA in MRSA was conducted as previously reported [43]. First, biofilm was cultured in a 6-well plate, the OD_600_ was measured by enzyme-labeled instrument, and the eDNA was extracted. The concentration of eDNA was measured with an DNA/Protein Analyzer (Pultton, USA). The relative content of eDNA was measured in terms of eDNA content per unit of biofilm.

### qRT-PCR

The extraction of total RNA was performed using the Simply P Total RNA Extraction Kit (Bioflux, Hangzhou, China). The cDNAs were synthesized by the PrimeScript™ RT reagent Kit with gDNA Eraser (Perfect Real Time) (Takara, Japan). The quantitative real-time polymerase chain reaction (qRT-PCR) was conducted using QuantStudio (Thermo Fisher, USA) with TB Green® Premix Ex Taq™ II (Takara, Japan). The primers and genes are listed in Table 3.

**Table 3:** q-RT-PCR Primers

Primer	Sequence (5′ → 3′)	Length (bp)
pyrB-F	AGGCATGGGCTTGCAGAAGAAAC	23
*pyrB-R*	CTCTATTCACAGGTGCCGGATGC	23
pyrF-F*pyrF-F*	ATGGCGTTGTTTGTTCACCTCTTG	24
*pyrF-R*	CGGTGTCGTAATACGGTGTTGGTC	24
*carB-F*	TTAACGTGCCACAGCCACAAGG	22
*carB-R*	TTCCATTGCGCGACCACCTAATAC	24
*arcC-F*	CATGCGGTGGTGGCGGTATTC	21
*arcC-R*	GGTATCTGCTTCAATCAGCGTTGC	24
*deoD-F*	TTACGCTGGTGTCGAAGCATTAGG	24
*deoD-R*	CCCTTTCCTCAGGTGTTGTTGACG	24
16S-F	GCTCGTGTCGTGAGATGTTGG	21
16S-R	TTTCGCTGCCCTTTGTATTGT	21

### Statistical analyses

All data except RNA-seq and metabolomics were reported as means ± SD. The significance of differences was determined by t-test using SPSS 23.0 (SPSS Inc.). A value of *P* < 0.05 was considered to be significant.

## Data Availability

The datasets generated during or analysed during the current study are available from the corresponding author on reasonable request. The datasets generated and/or analysed during the current study are available in the GEO repository, https://www.ncbi.nlm.nih.gov/geo/query/acc.cgi?acc=GSE157638 and GSE157638.

## References

[1] Firenzuoli F, Jaitak V, Horvath G, Bassolé IHN, Setzer WN, Gori L (2014). Essential oils: new perspectives in human health and wellness. Evid Based Complement Alternat Med.

[2] Zhao X, Liu Z, Liu Z, Meng R, Shi C, Chen X, Bu X, Guo N (2018). Phenotype and RNA-seq-based transcriptome profiling of Staphylococcus aureus biofilms in response to tea tree oil. Microb Pathog.

[3] Li W-R, Shi Q-S, Liang Q, Xie X-B, Huang X-M, Chen Y-B: Antibacterial Activity and Kinetics of *Litsea cubeba* Oil on *Escherichia coli*. PLoS One 2014;9(11):e110983. 10.1371/journal.pone.0110983 PMC4220960 25372706

[4] Rodriguez-Garcia I, Silva-Espinoza BA, Ortega-Ramirez LA, Leyva JM, Siddiqui MW, Cruz-Valenzuela MR, Gonzalez-Aguilar GA, Ayala-Zavala JF (2015). Oregano essential oil as an antimicrobial and antioxidant additive in food products. Crit Rev Food Sci Nutr.

[5] Rajkowska K, Nowicka-Krawczyk P, Kunicka-Styczyńska A: Effect of Clove and Thyme Essential Oils on Candida Biofilm Formation and the Oil Distribution in Yeast Cells. Molecules 2019;24(10):1954. 10.3390/molecules24101954 PMC6572016 31117281

[6] Carson CF, Hammer KA, Riley TV: Melaleuca alternifolia (tea tree) oil: a review of antimicrobial and other medicinal properties. Clin Microbiol Reviews 2006, 19(1):50. 10.1128/CMR.19.1.50-62.2006 PMC1360273 16418522

[7] Yadav E, Kumar S, Mahant S, Khatkar S, Rao R (2016). Tea tree oil: a promising essential oil. J Essent Oil Res.

[8] Organisation IS: Essential oil of Melaleuca, terpinen-4-ol type (Tea Tree oil). In: Huile essentielle de Melaleuca, terpinen-4-ol type (Huile essentielle de Tea tree) Geneva; 2017.

[9] Cordeiro L, Figueiredo P, Souza H, Sousa A, Andrade-Júnior F, Medeiros D, Nóbrega J, Silva D, Martins E, Barbosa-Filho J *et al*: Terpinen-4-ol as an Antibacterial and Antibiofilm Agent against *Staphylococcus aureus*. Int J Mol Sci. 2020;21(12):4531. 10.3390/ijms21124531 PMC7350221 32630600

[10] Lakhundi S, Zhang K: Methicillin-Resistant*staphylococcus aureus*: Molecular Characterization, Evolution, and Epidemiology. Clin Microbiol Rev. 2018;31(4):e00020-18. 10.1128/CMR.00020-18 PMC6148192 30209034

[11] Otto M (2013). Community-associated MRSA: what makes them special?. Int J Med Microbiol.

[12] Frieri M, Kumar K, Boutin A (2017). Antibiotic resistance. J Infect Public Health.

[13] Del Pozo JL (2017). Biofilm-related disease. Expert Rev Anti-Infect Ther.

[14] Mah TF (2012). Biofilm-specific antibiotic resistance. Future Microbiol.

[15] Katrin Schilcher and Alexander R Horswill: Staphylococcal Biofilm Development: Structure, Regulation, and Treatment Strategies. Microbiol Mol Biol Rev. 2010;84(3):e00026-19. 10.1128/MMBR.00026-19 PMC7430342 32792334

[16] Idrees M, Sawant S (2021). Nazira Karodia, and Ayesha Rahman*: Staphylococcus aureus biofilm: morphology, genetics, Pathogenesis and Treatment Strategies. Int J Environ Res Public Health.

[17] Brun P, Bernabe G, Filippini R, Piovan A (2019). In vitro antimicrobial activities of commercially available tea tree (Melaleuca alternifolia) essential oils. Curr Microbiol.

[18] Li Z, Wang N, Wei Y, Zou X, Jiang S, Xu F, Wang H, Shao X (2020). Terpinen-4-ol enhances disease resistance of postharvest strawberry fruit more effectively than tea tree oil by activating the Phenylpropanoid metabolism pathway. J Agric Food Chem.

[19] Li Y, Shao X, Xu J, Wei Y, Xu F, Wang H (2017). Tea tree oil exhibits antifungal activity against Botrytis cinerea by affecting mitochondria. Food Chem.

[20] Loughlin R, Gilmore BF, McCarron PA, Tunney MM (2008). Comparison of the cidal activity of tea tree oil and terpinen-4-ol against clinical bacterial skin isolates and human fibroblast cells. Lett Appl Microbiol.

[21] G. A. Pankey LDS: Clinical relevance of bacteriostatic versus bactericidal mechanisms of action in the treatment of gram-positive bacterial infections. Clin Infect Dis 2004, 38(6):864–870. 10.1086/381972 14999632

[22] Bordini EAF, Tonon CC, Francisconi RS, Magalhaes FAC, Huacho PMM, Bedran TL, Pratavieira S, Spolidorio LC, Spolidorio DP (2018). Antimicrobial effects of terpinen-4-ol against oral pathogens and its capacity for the modulation of gene expression. Biofouling.

[23] Maquera-Huacho PM, Tonon CC, Correia MF, Francisconi RS, Bordini EAF, Marcantonio E, Spolidorio DMP (2018). In vitro antibacterial and cytotoxic activities of carvacrol and terpinen-4-ol against biofilm formation on titanium implant surfaces. Biofouling.

[24] Majerczyk CD, Dunman PM, Luong TT, Lee CY, Sadykov MR, Somerville GA, Bodi K, Sonenshein AL (2010). Direct targets of CodY in Staphylococcus aureus. J Bacteriol.

[25] Pohl K, Francois P, Stenz L, Schlink F, Geiger T, Herbert S, Goerke C, Schrenzel J, Wolz C (2009). CodY in Staphylococcus aureus: a regulatory link between metabolism and virulence gene expression (vol 191, pg 2953, 2009). J Bacteriol.

[26] Rivera FE, Miller HK, Kolar SL, Stevens SM, Shaw LN (2012). The impact of CodY on virulence determinant production in community-associated methicillin-resistant Staphylococcus aureus. Proteomics.

[27] Montgomery CP, Boyle-Vavra S, Roux A, Ebine K, Sonenshein AL, Daum RS (2012). CodY deletion enhances in vivo virulence of community-associated methicillin-resistant Staphylococcus aureus clone USA300. Infect Immun.

[28] Majerczyk CD, Sadykov MR, Luong TT, Lee C, Somerville GA, Sonenshein AL (2008). Staphylococcus aureus CodY negatively regulates virulence gene expression. J Bacteriol.

[29] Desvaux M, Das T, Sehar S, Koop L, Wong YK, Ahmed S, Siddiqui KS, Manefield M: Influence of Calcium in Extracellular DNA Mediated Bacterial Aggregation and Biofilm Formation. PLoS One. 2014;9(3) :e91935. 10.1371/journal.pone.0091935 PMC3961253 24651318

[30] Qin Z, Ou Y, Yang L, Zhu Y, Tolker-Nielsen T, Molin S, Qu D (2007). Role of autolysin-mediated DNA release in biofilm formation of Staphylococcus epidermidis. Microbiology.

[31] Bonora M, Patergnani S, Rimessi A, De Marchi E, Suski JM, Bononi A, Giorgi C, Marchi S, Missiroli S, Poletti F (2012). ATP synthesis and storage. Purinergic Signalling.

[32] McCarthy H, Rudkin JK, Black NS, Gallagher L, O'Neill E, O'Gara JP. Methicillin resistance and the biofilm phenotype in Staphylococcus aureus. Front Cell Infect Microbiol. 2015;5:1. 10.3389/fcimb.2015.00001 PMC4309206 25674541

[33] Fernandez L, Hancock REW (2012). Adaptive and mutational resistance: role of Porins and efflux pumps in drug resistance. Clin Microbiol Rev.

[34] Abouelhassan Y, Zhang Y, Jin S, Huigens RW (2018). Transcript profiling of MRSA biofilms treated with a halogenated Phenazine eradicating agent: a platform for defining cellular targets and pathways critical to biofilm survival. Angew Chem Int Ed.

[35] Xu J, Shao X, Wei Y, Xu F, Wang H. iTRAQ proteomic analysis reveals that metabolic pathways involving energy metabolism are affected by tea tree oil in Botrytis cinerea. Front Microbiol. 2017;8:1989. 10.3389/fmicb.2017.01989 PMC5643485 29075250

[36] Marinus MG, Garavaglia M, Rossi E, Landini P: The Pyrimidine Nucleotide Biosynthetic Pathway Modulates Production of Biofilm Determinants in *Escherichia coli*. PLoS One. 2012;7(2):e31252. 10.1371/journal.pone.0031252 PMC3281075 22359582

[37] White AP, Gibson DL, Kim W, Kay WW, Surette MG (2006). Thin aggregative fimbriae and cellulose enhance long-term survival and persistence of Salmonella. J Bacteriol.

[38] Gualdi L, Tagliabue L, Bertagnoli S, Ieranò T, De Castro C, Landini P (2008). Cellulose modulates biofilm formation by counteracting curli-mediated colonization of solid surfaces in Escherichia coli. Microbiology.

[39] Kevin A (1998). Hughes IWSaMVJ: biofilm susceptibility to bacteriophage attack: the role of phage-borne polysaccharide depolymerase. Microbiology.

[40] Karaolis DKR, Rashid MH, Chythanya R, Luo W, Hyodo M, Hayakawa Y (2005). C-di-GMP (3′-5′-cyclic Diguanylic acid) inhibits Staphylococcus aureus cell-cell interactions and biofilm formation. Antimicrob Agents Chemother.

[41] Stepanović S, Vuković D, Hola V (2007). Quantification of biofilm in microtiter plates: overview of testing conditions and practical recommendations for assessment of biofilm production by staphylococci[J]. APMIS.

[42] Wen B , Mei Z , Zeng C , et al. metaX: a flexible and comprehensive software for processing metabolomics data. BMC Bioinformatics. 2017;18(1) :183. 10.1186/s12859-017-1579-y PMC5361702 28327092

[43] Rice KC, Mann EE, Endres JL (2017). The cidA murein hydrolase regulator contributes to DNA release and biofilm development in Staphylococcus aureus. Proc Natl Acad Sci U S A.

